# S-Adenosyl-Homocysteine Is a Weakly Bound Inhibitor for a Flaviviral Methyltransferase

**DOI:** 10.1371/journal.pone.0076900

**Published:** 2013-10-09

**Authors:** Hui Chen, Bing Zhou, Matthew Brecher, Nilesh Banavali, Susan A. Jones, Zhong Li, Jing Zhang, Dilip Nag, Laura D. Kramer, Arun K. Ghosh, Hongmin Li

**Affiliations:** 1 Wadsworth Center, New York State Department of Health, Albany, New York, United States of America; 2 Departments of Chemistry and Medicinal Chemistry, Purdue University, West Lafayette, Indiana, United States of America; 3 Department of Biomedical Sciences, School of Public Health, State University of New York, Albany, New York, United States of America; University of Texas Medical Branch, United States of America

## Abstract

The methyltransferase enzyme (MTase), which catalyzes the transfer of a methyl group from S-adenosyl-methionine (AdoMet) to viral RNA, and generates S-adenosyl-homocysteine (AdoHcy) as a by-product, is essential for the life cycle of many significant human pathogen flaviviruses. Here we investigated inhibition of the flavivirus MTase by several AdoHcy-derivatives. Unexpectedly we found that AdoHcy itself barely inhibits the flavivirus MTase activities, even at high concentrations. AdoHcy was also shown to not inhibit virus growth in cell-culture. Binding studies confirmed that AdoHcy has a much lower binding affinity for the MTase than either the AdoMet co-factor, or the natural AdoMet analog inhibitor sinefungin (SIN). While AdoMet is a positively charged molecule, SIN is similar to AdoHcy in being uncharged, and only has an additional amine group that can make extra electrostatic contacts with the MTase. Molecular Mechanics Poisson-Boltzmann Sovation Area analysis on AdoHcy and SIN binding to the MTase suggests that the stronger binding of SIN may not be directly due to interactions of this amine group, but due to distributed differences in SIN binding resulting from its presence. The results suggest that better MTase inhibitors could be designed by using SIN as a scaffold rather than AdoHcy.

## Introduction

Members of the Flavivirus genus, such as Dengue virus (DENV), Yellow Fever virus (YFV), West Nile virus (WNV), Tick-borne encephalitis virus (TBEV), and Japanese encephalitis virus (JEV) are ss-RNA (+) arthropod-borne viruses that can cause serious human disease, including meningitis, myelitis, encephalitis, and hemorrhagic fever [1–3]. Flavivirus infections are endemic to all continents except Antarctica. These viruses infect more than 200 million people and result in more than 100,000 fatalities per year [3]. Although effective vaccines exist for YFV, JEV, and TBEV [3] the difficulty of vaccinating large at-risk populations and the danger of adverse vaccination effects highlight the importance of developing antiviral therapeutics for treatment of severe flavivirus infections.

The flavivirus methyltransferase (MTase) has become an attractive target for such therapeutic interventions [4–16]. The flavivirus MTase, encoded by the NS5 gene, functions similarly to many other MTases to transfer a methyl group from its cellular cofactor molecule, S-adenosyl-methionine (AdoMet), first to the guanine-N-7 and then the ribose 2’-O of the flavivirus mRNA cap, with S-adenosyl homocysteine (AdoHcy) formed as a by-product in both steps [17–21]. Recently, the flavivirus MTase was also found to catalyze additional 2’-O methylations of internal adenosine of the viral RNA [22]. The first methylation of the viral mRNA cap is an obligate step in the virus life-cycle; and defects in N-7 methylation are lethal to DENV, WNV, YFV, and Kunjin virus replication [18,19,21,23–26]. Our laboratory recently identified an AdoMet analogue, sinefungin (SIN) that inhibits the MTase activity and replication among a broad spectrum of flaviviruses [4,23]. We also observed an additional pocket adjacent to the AdoMet/SIN/AdoHcy binding site; this pocket is specific to and conserved among flavivirus MTase but not found in human MTases [23].

A series of highly selective AdoHcy-based inhibitors of the flavivirus Mtase, that did not inhibit human Mtases, were recently reported to target this pocket, although the antiviral efficacy of the compounds was characterized [15]. To investigate whether more potent and selective inhibitors of the flavivirus MTase could be identified, we designed and synthesized four new AdoHcy derivatives. Unfortunately, these derivatives did not show improved activity towards the viral MTase activity. Upon examination of the intrinsic inhibitory ability of AdoHcy, we unexpectedly found that AdoHcy barely inhibits the N-7 and 2’-O activities of the flavivirus MTase, even at high concentrations. We further observed that AdoHcy also does not inhibit virus growth in cell-culture. Binding studies showed that AdoHcy has a much lower binding affinity than AdoMet and SIN. This result is consistent with computational Molecular Mechanics Poisson-Boltzmann surface Area (MM-PBSA) analysis indicating that SIN has a more favorable binding free energy with the MTase than AdoHcy. Our results indicated that SIN might be a better scaffold to design new inhibitors as compared to AdoHcy.

## Results

### Synthesis of AdoMet analogs

**Figure 1:**
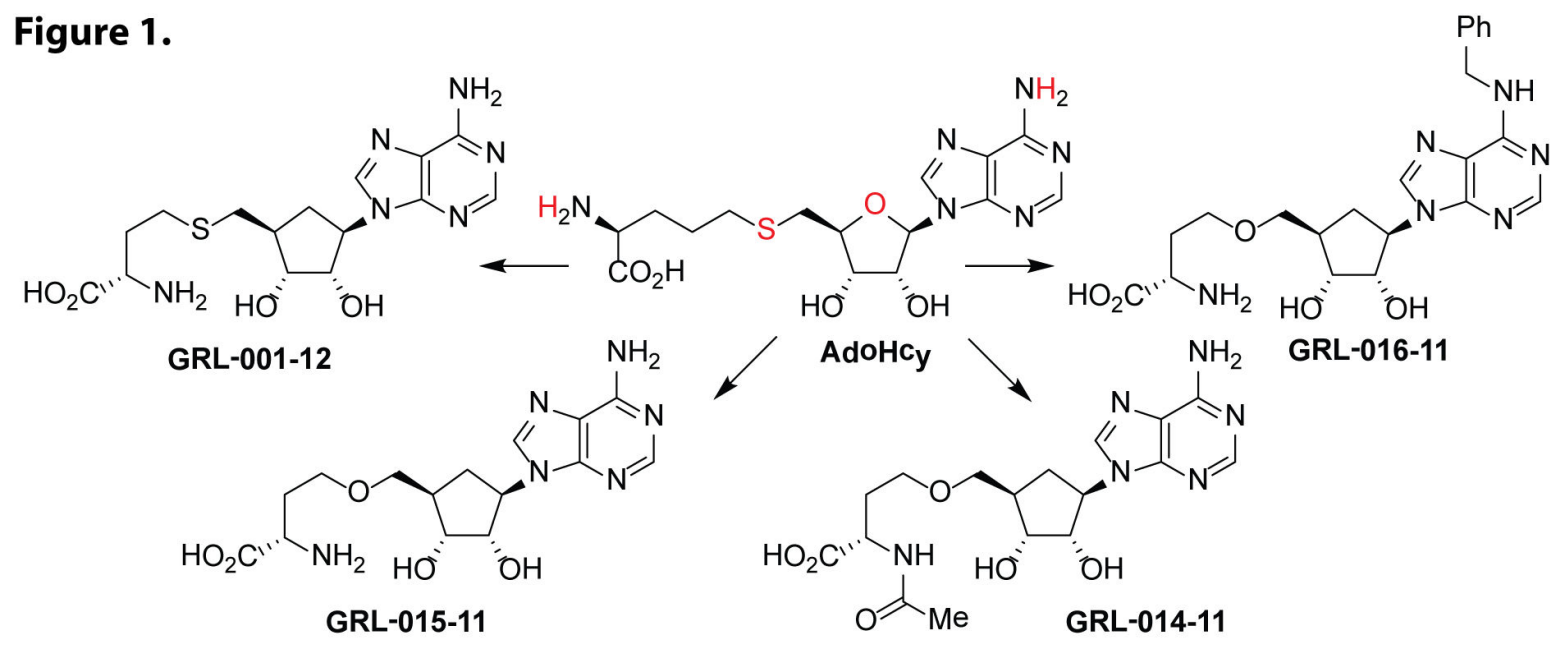
Chemical structures of AdoHcy and its four derivatives synthesized. Groups of AdoHcy that were modified were in red color.

We have previously found a natural product, sinefungin (SIN), and several nucleoside analogs inhibited both the MTase activities *in vitro*, as well as virus growth in cell culture [4,16,23]. The AdoHcy by-product of the MTase reaction and a number of its derivatives were also found to be potent MTase inhibitors in several studies [8,14,15]. In order to obtain more specific and potent inhibitors against flavivirus MTase, we designed and synthesized four AdoHcy derivatives (Figure **1**). These analogs were chosen rationally, based upon the inhibitor-bound MTase structures [15,23]. In particular, we plan to fill in the conserved hydrophobic pocket present in flavivirus MTases [15,23] and to stabilize the sugar and amino parts of the analog (Figure **1** and Figure **S1**).

**Figure 2:**
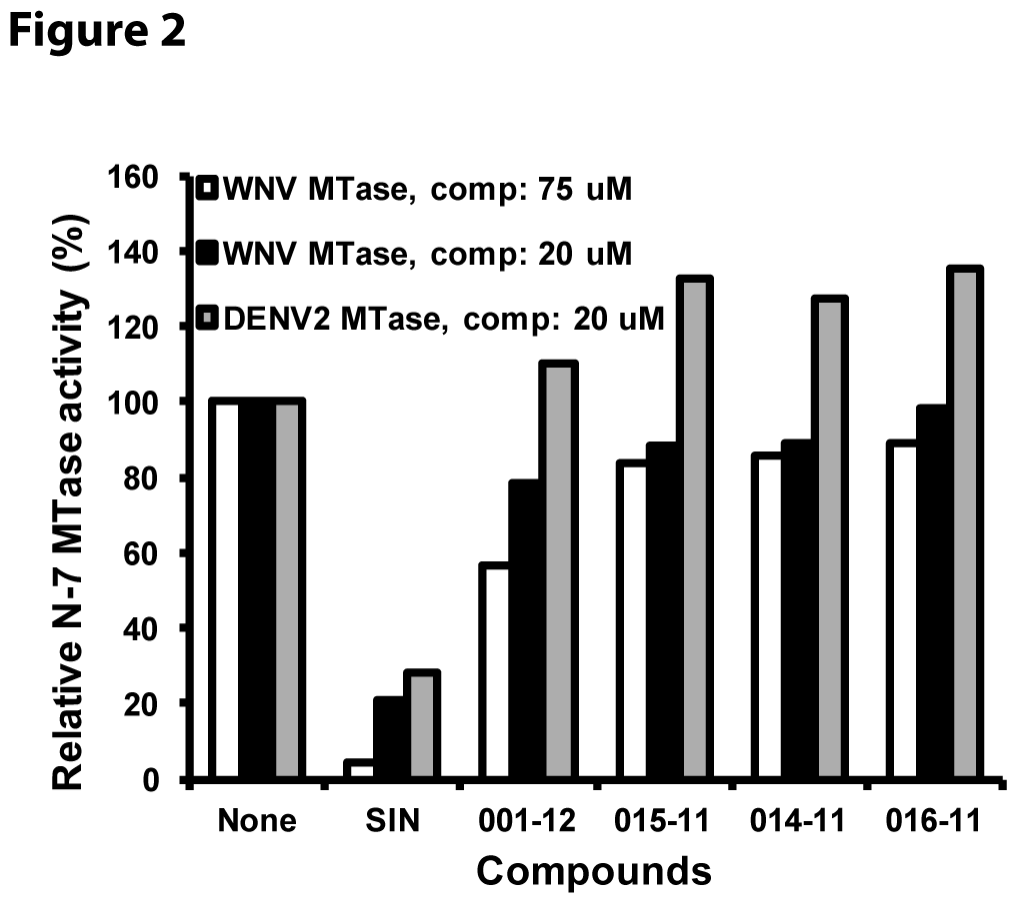
Inhibition of the N-7 activities of the WNV and DENV2 MTases by synthesized derivatives at 20 µM or 75 µM concentrations. The methylation activity without compounds was set at 100%. Details were described in the legend for Figure **3**.

Used the WNV MTase as a model, we first measured the N-7 MTase activity of the WNV MTase in the presence of the four compounds at both 20 µM and 75 µM concentrations. We added SIN as a positive control. As shown in Figure **2**, the positive control inhibitor SIN efficiently inhibited (~80%) the N-7 activity of both DENV2 and WNV MTases at 20 µM concentration. Increasing the concentration of SIN to 75 µM almost completely abolished the N-7 activity of the WNV MTase. This dose-dependent inhibition by SIN validated the effectiveness of our assay. As shown in Figure **2**, the four AdoHcy-derivatives barely showed any inhibition of the N-7 MTase activity of the WNV MTase at 20 µM concentration. For the DENV2 MTase, these compounds even enhanced the N-7 MTase activity, due to unknown reasons. On increasing the concentration of compounds to 75 µM, they reduced the WNV N-7 MTase activity by less than 20% (Figure **2**). The only exception was compound **GRL-001-12**, which reduced the WNV N-7 MTase activity by about 44% at this higher concentration.

### AdoHcy does not inhibit the N-7 and 2’-O MTase activities of flavivirus MTases

**Figure 3:**
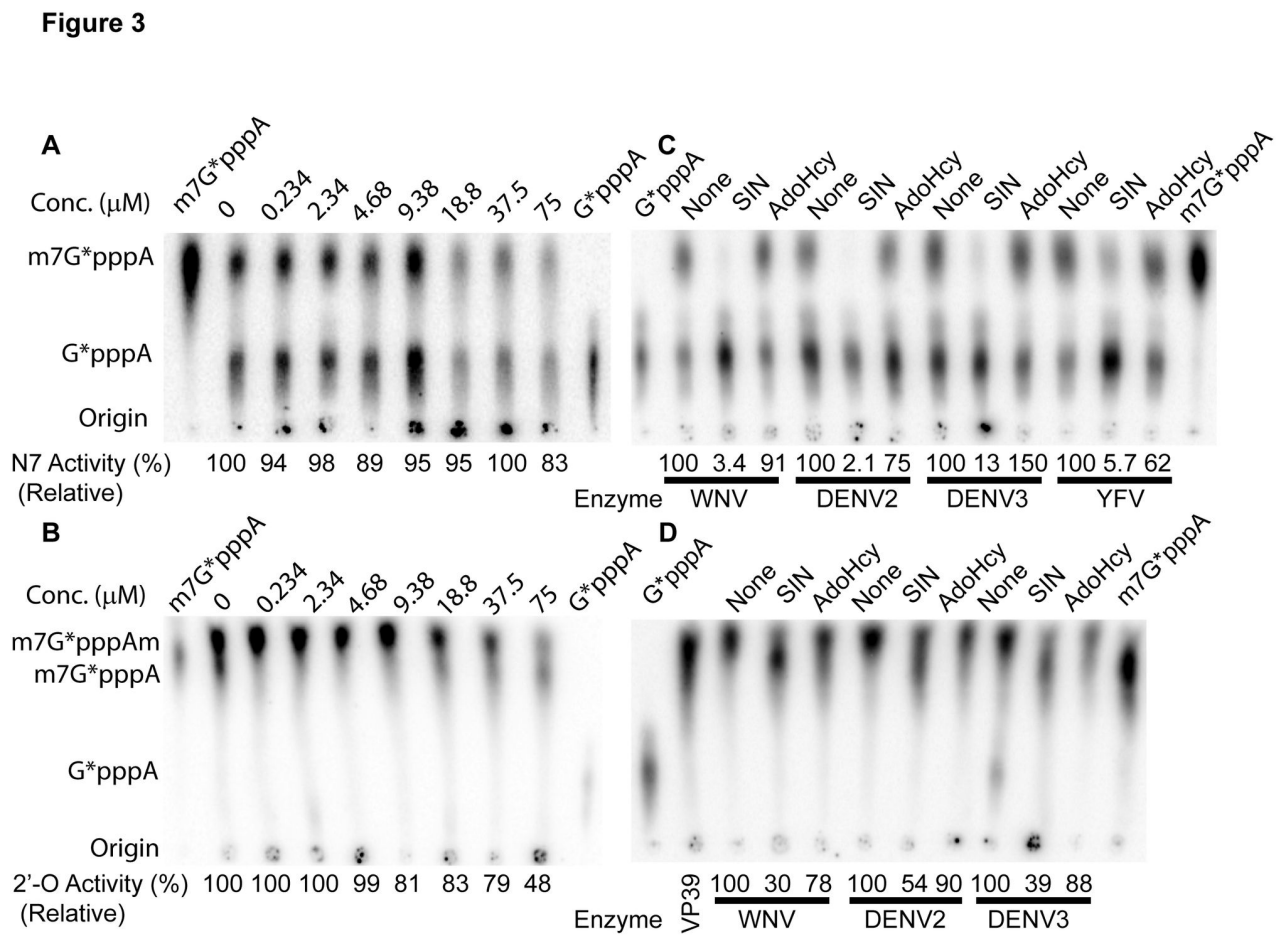
Inhibition of the N-7 and 2’-O methylation activities of the flavivirus MTases by AdoHcy. (**A**) TLC analysis of inhibition of the N-7 methylation activity of the WNV MTase by AdoHcy was analyzed on TLC plates. The spots representing different cap structures on TLC plates were quantified by a PhosphorImager. The N-7 methylation was measured by conversion of G*pppA-RNA→m^7^G*pppA-RNA (e.g., the specific activity (%) = Intensity (m^7^G*pppA)/(Intensity (G*pppA) +Intensity (m^7^G*pppA)) *100) (Here and after, the asterisk indicates that the following phosphate is ^32^P labeled; the RNA represents the first 90 nucleotides of the WNV genome). The relative methylation activity without AdoHcy was set at 100%, and the relative methylation activity with a particular compound was defined as specific activity (compound)/specific activity (no compound) * 100. (**B**) TLC analysis of inhibition of the 2’-O methylation activity of the WNV MTase by AdoHcy. The 2’-O methylation was measured by conversion of m^7^G*pppA-RNA→m^7^G*pppAm-RNA (e.g., the specific activity (%) = Intensity (m^7^G*pppAm)/(Intensity (m^7^G*pppA) +Intensity (m^7^G*pppAm)) *100). The methylation activity without AdoHcy was set at 100%, and the relative 2’-O methylation activity with compounds was defined the same way as in panel A. The migration positions of the G*pppA and m^7^G*pppA molecules are labeled on the side of the TLC images. (**C**-**D**) TLC analysis of inhibition of the N-7 (**C**) and 2’-O (**D**) methylation activities of flavivirus MTases in the presence or absence of 150 µM SIN or 150 µM AdoHcy. The methylation activity for each MTase without compound was set at 100%. The relative methylation activity for each MTase with compound (SIN or AdoHcy) was calculated as percentage to the activity without any compound. The migration positions of the G*pppA, m^7^G*pppA, and m^7^G*pppAm molecules are labeled on the side of the TLC images. VP39 was included in Panel **D** as a positional control for the 2’-O methylation reaction.

The lack of inhibitory activity in these analogs was quite surprising, particularly because these compounds are very close derivatives of AdoHcy, which showed high potency in inhibition of the MTase activities in a number of studies [8,14,15,27]. These results prompted us to investigate whether AdoHcy is an effective inhibitor for flavivirus MTase. We performed the MTase inhibition assay for a series of concentrations of AdoHcy. Our results showed that, even at a high concentration of 75 µM, AdoHcy did not inhibit the N-7 activity of the WNV MTase (Fig. **3A**), and only inhibited 52% of the 2’-O activity of the WNV MTase (Fig. **3B**). For the 2’-O reactions, in contrast to our previous observations [4,18,19,23,24,28], the methylated product migrated to a position higher than the mono-methylated m7GpppA. The material migrating to the higher position was confirmed to be double methylated m^7^GpppAm, since the double methylated m^7^GpppAm product converted from m^7^GpppA by a control cap-dependent 2’-O MTase VP39 migrated to the same position (Fig. **3D**). As reported in a recent study [29], this change might have been caused by the use of nuclease P1 from SIGMA-Aldrich, instead of the nuclease P1 from US Biological used in all our previous experiments. Nevertheless, the results indicated that AdoHcy, the by-product of the MTase reactions, is not an inhibitor for the N-7 activity of the WNV MTase and only a weak inhibitor for the 2’-O MTase activity.

To determine whether other flavivirus MTases can be inhibited by AdoHcy, we expressed and purified the YFV MTase as described previously [19,28]. We also cloned, expressed, and purified the DENV2 and DENV3 MTase domains in bacteria, either as a His-tag fusion protein (DENV2) or as a GST-tag fusion protein (DENV3). The tag-free DENV3 MTase was purified by removal of the GST-tag through PreScission protease digestion, followed by gel filtration chromatography. We used the WNV RNA, containing the 5’-terminal 90 nucleotides of the genome, to assay for methylation activities of the four MTases. The substrate was known to react with MTases from other flaviviruses such as DENV, YFV, and Powassan virus [19,28]. As expected, in the absence of inhibitors, MTases from DENV2, DENV3, or YFV could efficiently methylate the WNV G*pppA-RNA at the N-7 position, reaching 53 to 116% of the WNV MTase activity (Figure **3C**). For the 2’-O MTase activity in the absence of inhibitors, both DENV2 and DENV3 MTases could effectively methylate more than 95% of the WNV m^7^G*pppA-RNA substrate to m^7^G*pppAm-RNA (Figure **3D**), whereas the YFV MTase failed to methylate the WNV substrate at the 2’-O position. These negative results for 2’-O methylation by the YFV MTase are not shown, but similar results have been reported previously [19,28]. Since flavivirus MTase is known to require distinct RNA elements for methylations [28], it is possible that the WNV RNA substrate used is not optimal for 2’-O methylation by the YFV MTase.

To simplify the calculations, we set the MTase activity to 100% for each MTase in the absence of inhibitors, and then calculated the relative activity for each MTase in the presence of inhibitor as percentage to that without inhibitor. As expected, in the presence of 150 µM concentration of SIN, the N-7 activities of all four MTases were almost completely abolished (Figure **3C**), and the 2’-O activities were significantly inhibited by 70%, 46%, and 61% for the WNV, DENV2, and DENV3 MTases, respectively (Figure **3D**). In contrast, in the presence of 150 µM of AdoHcy, the WNV, DENV2, DENV3, and YFV MTases could still respectively methylate 91%, 75%, 150%, and 62% of the G*pppA-RNA substrate at the N-7 position; similarly the m7G*pppA-RNA substrate could be methylated to 78%, 90%, and 88% at the 2’-O position by the WNV, DENV2, and DENV3 MTases, respectively. These results indicated that AdoHcy either does not or only very weakly inhibits the N-7 and 2’-O MTase activities of flavivirus MTases.

### Cytotoxicity and antiviral analyses

**Figure 4:**
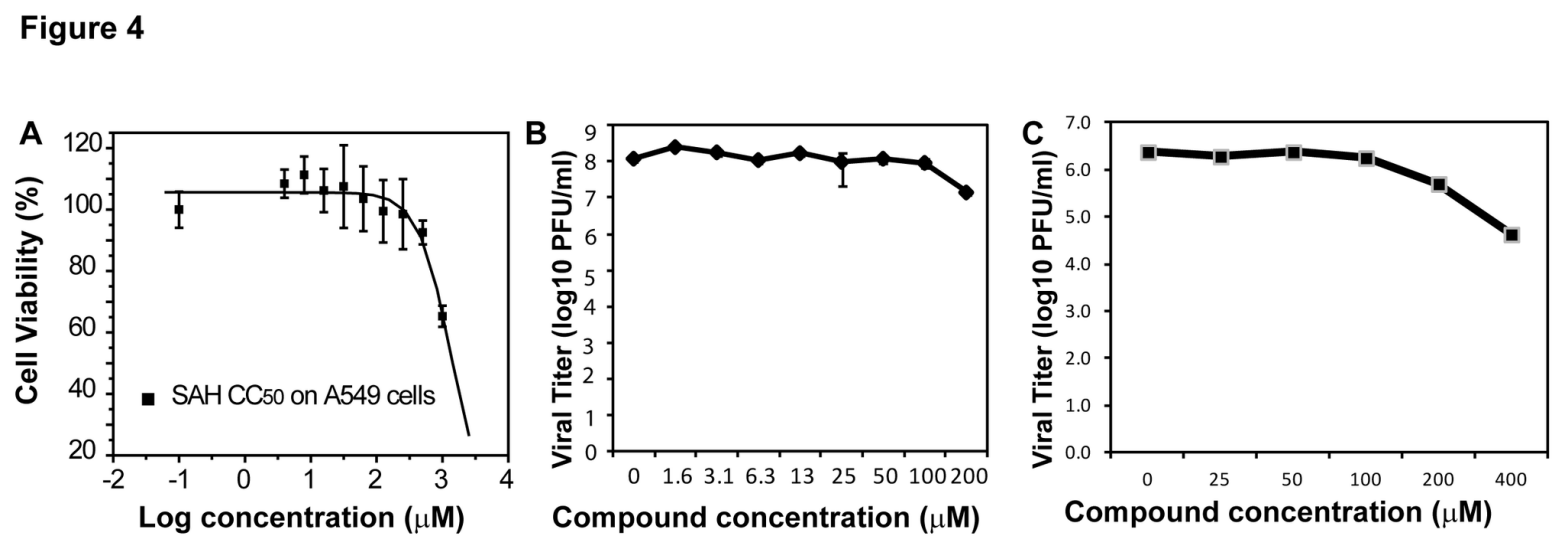
Cytotoxicity and antiviral analyses for AdoHcy. (**A**) Cytotoxicity of AdoHcy. A549 cells were incubated with various concentrations of AdoHcy and then assayed for viability at 42 h postincubation. (**B**) Inhibition of the WNV replication by AdoHcy. (**C**) Inhibition of the DENV2 replication by AdoHcy. A549 cells were infected with WNV or DENV2 at an multiplicity of infection of 0.1, in the presence or absence of AdoHcy. At 42 h post-infection, viral titers in culture fluids were quantified by plaque assays on Vero cells.

Although AdoHcy does not inhibit the flavivirus MTase activity *in vitro*, it may inhibit virus growth *in vivo*. To rule out this possibility, we performed cell-based assays to evaluate the biological activities of AdoHcy. We first used a MTT cell proliferation assay to measure the cytotoxicity of AdoHcy to a human A549 cell line (Figure **4A**). Our results indicated that AdoHcy did not show cytotoxicity at 0.5 mM concentration. Even at 1 mM concentration of AdoHcy, the cells remained 65% viability.

We next performed viral titer reduction assay to evaluate the compound antiviral efficacy. As shown in Figure **4**, AdoHcy did not inhibit the growth of DENV2 virus and only inhibited about 50% of the WNV growth at 100 µM concentration. At 200 µM concentration, AdoHcy could effectively inhibit the growth of DENV2 (Figure **4C**) and only reduced the viral titer of WNV by approximately one log order (Figure **4B**). Therefore, the EC_50_ values of AdoHcy for DENV2 and WNV were estimated to be over 100 µM (EC_50_, effective concentration of a compound required to inhibit 50% of virus growth). Overall, our results indicated that AdoHcy only inhibited virus growth at very high concentration.

### AdoHcy has lower binding affinity for flavivirus MTase

In order to understand why SIN but not AdoHcy can inhibit the MTase activities, we developed an AdoMet-binding assay. For this assay, biotinylation of the WNV and DENV3 MTases was required. Upon biotinylation, the WNV MTase became insoluble and precipitated from solution, while the DENV3 MTase remained soluble. We therefore mixed the biotinylated DENV3 MTase with streptavidin-coated SPA beads (PerkinElmer). Binding of [^µ^H] AdoMet to the biotinylated-MTase attached to the beads triggered the beads to emit light which was monitored by a Microbeta^2^ plate counter.

**Figure 5:**
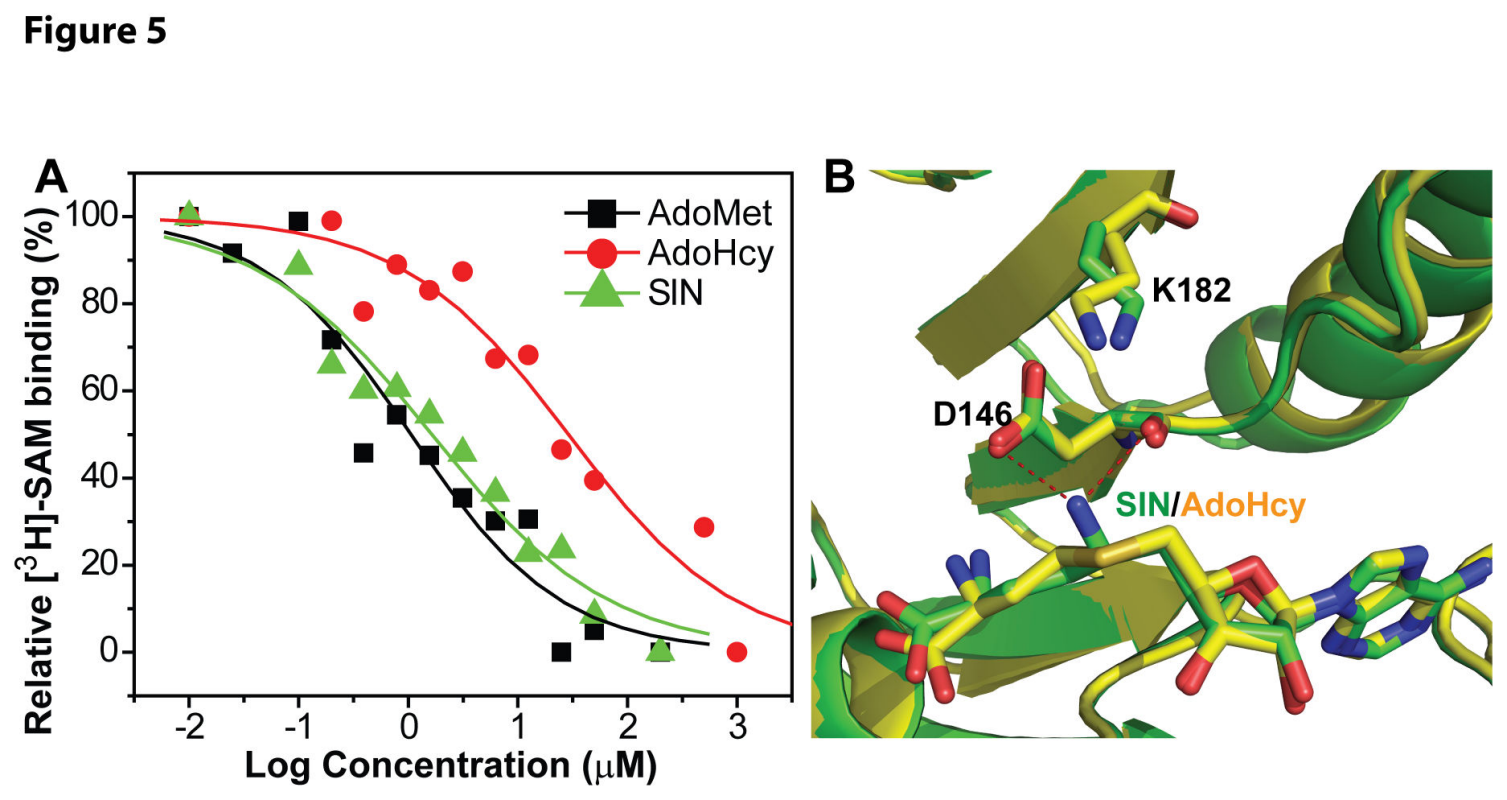
AdoHcy binds the DENV3 MTase with a much weaker affinity than do AdoMet and SIN. (**A**) Dose response of inhibition of the [^3^H]-SAM-MTase complex formation by AdoMet (black), AdoHcy (red), and SIN (green). The biotinylated DENV3 MTase and ^3^H-labeled SAM were incubated with or without compounds AdoMet, AdoHcy, and SIN. A two-fold dilution series was shown for each compound. The reaction mixtures were mixed with the streptavidin-coated SPA beads and quantified using a Microbeta^2^ scintillation counter. (**B**). Superposition of the crystal structures of the MTase-SIN complex (green) [23] and the MTase-SAH complex (yellow) [19]. SAH and SIN are shown in stick representation. Atomic color coding is as follows (unless otherwise specified): carbon in yellow/green, oxygen in red, nitrogen in blue, and sulfur in orange. Potential hydrogen bonds are depicted in red dashed lines.

We examined the ability of the compounds to compete against ^3^H-labeled AdoMet-MTase complex formation (Figure **5**). Our data showed that AdoMet binds the DENV3 MTase with a high affinity with a *K*_*d*_ of 1.05 µM. SIN binds the MTase with an affinity of 1.64 µM, which is comparable to that of AdoMet. In contrast, AdoHcy binds the MTase with a much lower binding affinity (*K*_*d*_ = 28.9 µM) than do AdoMet and SIN. The affinity of AdoHcy for the MTase is 28-fold and 18-fold lower than those of AdoMet and SIN, respectively. Overall, this data indicated that AdoHcy has a much weaker binding affinity for flavivirus MTase.

### MM-PBSA analysis of AdoHcy and SIN binding to the WNV MTase

To better understand the detailed differences between AdoHcy and SIN binding to the flavivirus MTase, we performed MM-PBSA analysis of MD simulations of the two compounds bound to the WNV MTase. The WNV MTase was chosen since crystal structures for both AdoHcy and SIN bound to the WNV MTase are available at high resolution [19,23], and a crystal structure of the DENV3 MTase in complex with SIN has not been determined. The crystal structures of the flavivirus MTases are highly conserved, especially at the AdoMet-binding site [23,30], so the present analysis might be generally applicable to all other flavivirus MTases.

The SIN and AdoHcy molecules are especially comparable to one another since they differ by only a few atoms, and both bind in near identical orientations to the WNV MTase with the protein structure around them also remaining very similar (PDB codes: 2OY0 and 3LKZ [19,23]). Both ligands are also uncharged in solution, and the atoms that are chemically different between them are solvent-exposed in their complexes with the WNV MTase. This suggests that the difference in binding energy between the two ligands may not arise from a structural difference in the way they are bound, but from an underlying energetic reason. To assess this possibility, we performed explicit solvent simulations of both ligands bound to the WNV MTase, and assessed their absolute binding free energies using MM-PBSA analysis. The results of this analysis are shown in Table 1. While both ligands were predicted to bind strongly to the WNV MTase in the conformation corresponding to the crystal structure, the binding energy of SIN was estimated to be 6.8 kcal/mol more favorable than that of AdoHcy. The breakdown of this binding energy difference into vacuum interaction (-12.3 kcal/mol), electrostatic solvation (+8.9 kcal/mol), non-polar solvation (-0.7 kcal/mol), and solute entropic components (-2.7 kcal/mol) suggests that more favorable electrostatic and van der Waal’s interactions between SIN and the WNV MTase atoms are primarily responsible for differences in binding.

**Table 1:** Energetic analysis for AdoHcy and SIN binding to WNV MTase.

Binding	AdoHcy (kcal/mol)					SIN (kcal/mol)					Difference
Energy	Complex	Protein	Ligand	ΔG_b_		Complex	Protein	Ligand	ΔG_b_		ΔΔG_b_ (SIN-SAH)
MMVE	-1851.8	-1764.4	35.9	-123.3		-1752.0	-1627.5	11.1	-135.6		-12.3
PB	-1023.5	-1029.4	-10.1	16.0		-1032.0	-1047.2	-9.7	24.9		8.9
SA	81.3	81.9	4.3	-4.9		81.3	82.5	4.4	-5.6		-0.7
-TΔS	-223.2	-222.9	-24.0	23.7		-222.8	-222.5	-21.3	21.0		-2.7
Total	-3017.2	-2934.8	6.1	-88.5		-2925.5	-2814.7	-15.5	-95.2		-6.8

All values are in kcal/mol. The energetic components labels are as follows: MMVE: Molecular Mechanics vacuum energy; PB: Poisson-Boltzmann electrostatic solvation energy; SA: Surface Area based non-polar solvation energy; - TΔS: entropic contribution to total free energy based on quasi-harmonic estimation of solute entropy; ΔG_b_: binding energy component (Complex - Protein - Ligand).

**Figure 6:**
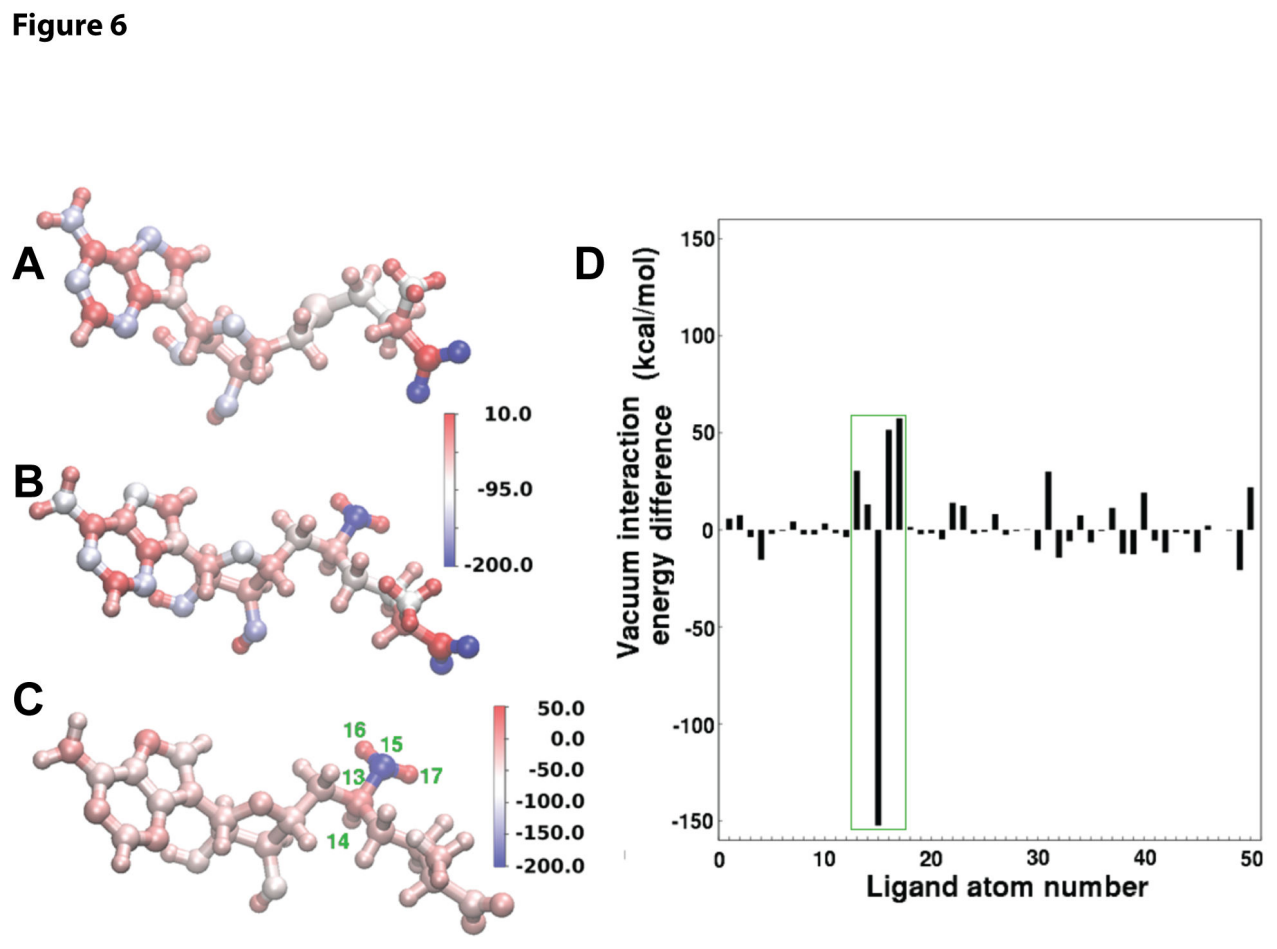
Atomic decomposition of the vacuum interaction energy between the AdoHcy and SIN ligands and the WNV MTase. Color coded projection onto the individual ligand atoms of the interaction energy between AdoHcy (panel **A**), SIN (panel **B**), and the difference (SIN-AdoHcy projected on SIN, panel **C**), and the WNV MTase. Panel **D** shows the numerical value of the interaction energy difference between the two ligands with the chemically different atoms bounded by a green box. The chemical difference is localized to atoms 13-17: atom 13 is a sulfur in AdoHcy, while atoms 13-17 are CHNH2 in SIN. The atoms numbers are indicated by green labels in panel C. All values are in kcal/mol.

The SIN and AdoHcy molecules are especially comparable to one another since they both bind in a very similar orientation to the WNV MTase (PDB codes: 2OY0 and 3LKZ [19,23]), differ by only a few atoms, and are both expected to be neutral in physiological conditions. The decomposition of the vacuum interaction energies into contributions from individual atoms in the AdoHcy and SIN ligands (Table **S1**), illustrated in Figure **6**, shows that the overall average difference of about -12 kcal/mol is not directly due to the chemically different atoms between AdoHcy and SIN. Although, the nitrogen in the extra NH2 group in SIN has highly attractive interactions with the protein (-152.4 kcal/mol), these are effectively cancelled by the repulsive interactions with its neighboring carbon and hydrogens (+152.2 kcal/mol). Instead, the interactions with the other chemically identical atoms in the two ligands are cumulatively biased towards a greater overall attractive interaction between SIN and the protein, possibly due to subtle alterations in the protein and ligand geometries in the presence of the NH2 group in SIN.

## Discussion

Many flaviviruses cause significant human disease. Unfortunately, specific antiviral therapy does not exist to date. Recently, flavivirus MTase became an attractive drug target due to its essential N-7 MTase function in viral replication [4–6,8–15,18,19,21,23,25,26,30]. AdoHcy, the by-product of the methyl transfer reaction, has been shown to inhibit both N-7 and 2’-O MTase activities for WNV, DENV2 and DENV3 [8,14,15,27]. The *IC*_*50*_ values for inhibition of the WNV and DENV3 MTase activities by AdoHcy were estimated to be in low micromolar or even nanomolar range (from 0.34 µM to 3.19 µM) (*IC*_*50*_: compound concentration required to inhibit enzyme activity by 50%). In contrast, our results showed that AdoHcy and its derivatives do not significantly inhibit both the N-7 and 2’-O activities for MTases from four different viruses (WNV, DENV2, DENV3, and YFV), even at very high concentrations (150 µM). Although there are some differences in assay conditions such as buffers, pH, substrates, and constructs of enzymes used, it is hard to believe that they will account for the large discrepancies. As shown in Figure **3**, in the absence of the positive control SIN inhibitor, all enzymes could efficiently carry out the N-7 and 2’-O MTase reactions in our experiments. Under the same conditions as for AdoHcy, SIN could efficiently abolish the MTase activities of all MTases from the four viruses, which is consistent with our previous results showing that SIN inhibited both MTase activities of the WNV MTase with *IC*_*50*_ about 14 µM using the TLC method [4]. In contrast, AdoHcy under the same conditions failed to inhibit the enzymatic activities (Figure **3**).

The discrepancies may more reasonably be attributed to the different methods used to monitor the reactions. We monitored the reaction product, m^7^G*pppA and double methylated m^7^G*pppAm (^32^P labeled phosphate following the asterisk is), using the TLC method [16,18,19,23]. Although this method is low throughput, its advantage is the ability to directly “visualize” and quantify the reaction product. Alternative higher throughput monitoring methods could possibly quantify non-specific binding of radiolabeled materials and/or signals arising from incorporation of radio-labeled materials to other positions of RNA. Previous studies employed the SPA-based scintillation assay in which [^3^H]-AdoMet was used as a co-factor and activity was monitored by scintillation counting of the transfer of [^3^H]-labeled methyl group to the viral RNA [8,14,15,27]. Non-specific binding of radio-labeled materials or incorporation of radio-labeled materials to positions other than N-7 and 2’-O of the RNA could affect the activity reported by this assay. It was reported that N-7 and 2’-O reactions might only account for one-third of the total signals and that a large fraction of signals were unresolved when using the SPA method [27]. In particular, the flavivirus MTase was reported to also carry out 2’-O methylation of internal adenosines in the viral RNA [22]. The unresolved signals therefore could be from methylations of internal adenosines of the RNA. The presence of these unresolved signals may thus affect how the results from inhibition studies using the SPA method were interpreted. It is possible that AdoHcy might mainly inhibit the internal methylation activity of flavivirus MTase, for which the hypothesis requires further investigation.

The weak inhibition of the N-7 and 2’-O activities of flavivirus by AdoHcy are consistent with functional analysis indicating that it does not suppress viral growth till a high concentration (200 µM) is reached (Figure **4**). In contrast, SIN inhibits both N-7 and 2’-O activities of the WNV MTase with *IC*_*50*_ of 14 µM *in vitro* [4], and can also efficiently inhibit the growth of WNV with an *EC*_*50*_ of 27 µM [4]. The ineffectiveness of AdoHcy in virus growth inhibition is also consistent with results from a number of studies showing that the circulating blood levels of AdoHcy are as high as 0.77 µM [31–35], and the levels of AdoMet are as high as 2.6 µM [31–35]. The binding affinity of AdoHcy for the DENV3 MTase was also shown to be much lower than those of AdoMet and SIN. The low affinity of AdoHcy for the MTase may facilitate the by-product release from the MTase and replenishment with a fresh AdoMet for a new cycle of methylation reaction.

Structural comparison also supports the results. Superposition of the crystal structures of the WNV MTase-SIN (PDB: 3LKZ) and MTase-AdoHcy (2OY0) complexes reveals that SIN binds to the AdoMet pocket of the MTase in a conformation similar to that of AdoHcy in the MTase-AdoHcy complex (Figure **5B**) [19,23]. However, the free amine NE of the C-NH2 group of SIN, i.e., the group that replaces the S-CH3 group of AdoMet, makes at least five additional contacts with the MTase, which include a pair of potential hydrogen bonds between the NE atom of SIN and the OD1 and O atoms of the MTase catalytically essential residue D146 (Figure **5B**). The structural results correlate very well with MM-PBSA analysis of binding of SIN and AdoHcy to the WNV MTase, which showed that SIN binds the WNV MTase more favorably than AdoHcy by 6.8 kcal/mol (Table 1), and that the NH2 group of SIN alone makes the largest contribution (Tables **1** and **S1**, Figure **6**). The binding free energy difference can also be estimated from the difference in binding constants for SIN and AdoHcy binding to the MTase using the equation: ΔΔG=-RT(InK_*d(SIN)*_)-(-RT(InK_*d(AdoHcy)*_), where R is gas constant, T is temperature in degree Kelvin, and *K*_d (*AdoHcy*)_ and *K*_*d* (*SIN*)_ are binding constants for AdoHcy and SIN binding to the MTase, respectively [36]. This binding free energy difference of -7.1 kcal/mol derived from experimental measurements is a very good agreement with the MM-PBSA estimate of -6.8 kcal/mol obtained from the MD simulations.

In summary, this study investigated the inhibition of an essential flavivirus MTase by the reaction by-product AdoHcy, its derivatives, and a natural inhibitor SIN. Our results demonstrated that the AdoHcy only weakly inhibits flavivirus MTases and had a much weaker binding affinity for flavivirus MTase than SIN and the co-factor AdoMet. Most importantly, the AdoHcy does not inhibit viral growth in cell culture until a high concentration, whereas the natural inhibitor SIN inhibits viral growth at much lower concentrations. Therefore, SIN rather than AdoHcy should be considered as a good structural scaffold for future development of inhibitors for MTases from flavivirus families, or even more broadly for development of AdoMet-based inhibitors for any AdoMet-utilizing enzymes, as seen in a recent report [37].

## Materials and Methods

### Compounds

Four nucleoside analogs were designed and synthesized in optically active form with defined stereochemistry (Figure **S1**). The details will be published elsewhere.

SIN and AdoHcy were purchased from Sigma-Aldrich. AdoMet was purchased from New England Biolabs. [3H] AdoMet was purchased from PerkinElmer. [α-32P]GTP was purchased from MP Biomedicals.

### Cloning, expression, and purification of the NS5 MTase from WNV, YFV, DENV2 and DENV3

Recombinant MTases from WNV, YFV, DENV2 and DENV3 contained the N-terminal 300, 266, 265, and 272 amino acids of NS5 protein, respectively. The MTases from WNV, YFV, and DENV2 had a His tag to facilitate purification: WNV MTase contained an N-terminal His tag, whereas DENV2 and YFV MTases had a C-terminal His tag. The DENV3 was produced using a GST-tag. The WNV and YFV MTases were prepared as described previously [18,19,28]. For cloning of DENV2 MTase, a DNA fragment representing the MTase domain (amino acid (aa) 1-296) was PCR amplified from the New Guinea C strain of DENV2 using a pair of primers CGCGGATCCAACATAGGAGAGACGCTTGGAGA and CCCAAGCTTCTATTGGTCATAGTGCCATGATGTTTC and was inserted into the pQE30 vector (Qiagen) at the *BamHI* and *HindIII* sites (underlined). To facilite stable crystallization, a shorter version of the DENV2 MTase (aa 1-265) was cloned into the the pET26b vector (EMD Biosciences) at the *NdeI* and *HindIII* sites using a pair of primers GCGGATCCCATATGACGGGAAACATAGGAGAGACGCTTGGAGAG and CCCAAGCTTCTAATGGTGGTGATGATGGTGTGAGCTTGATCCGATGTTGCGGGTTCCG (restriction sites were underlined). The shorter DENV2 MTase (aa 1-265) contained additional C-terminal SSSHHHHHH sequence according to the reported crystal structure [38] and was used throughout this manuscript.

To clone the DENV3 MTase domain (aa 1-272), a pair of primers CGCGGATCCGGAACGGGGTCACAAGGC and ATAGTTTAGCGGCCGCCTAGTTGGGTGTTTCTGGTTCCGC (restriction sites underlined) were used to PCR amplify the DENV3 MTase fragment from a DENV3 isolate from the UTMB virus collection. The PCR fragment was cloned into the pGEX-6P-1 vector (GE HealthCare) at the *BamHI* and *NotI* sites. The DENV2 and DENV3 MTases were expressed in *Escherichia coli* strain Rosetta 2(DE3) (EMD Biosciences) and purified through a nickel-nitrilotriacetic acid column (DENV2) or a glutathione Sepharose 4B column (DENV3). The affinity-purified DENV2 MTase was further purified by a gel filtration 16/60 Superdex column (GE HealthCare). The affinity-purified DENV3 MTase-GST fusion protein was digested with the PreScission protease according to the manufactory protocol, re-loaded to the glutathione column to remove the affinity GST-tag and residue undigested fusion protein, and further purified to homogeneity by a gel filtration 16/60 Superdex column (GE HealthCare).

### *In vitro* MTase inhibition assay

The 5’-end ^32^P-labeled substrates G*pppA-RNA and m^7^G*pppA-RNA, representing the first 90 nucleotides of the WNV genome (the asterisk indicates that the following phosphate is ^32^P labeled), were prepared as described previously [4,16,19]. The N-7 and 2'-O methylation inhibition assays were performed as described previously [16,18,19]. The N-7 methylation was measured by conversion of G*pppA-RNA→m^7^G* pppA-RNA. The 2’-O methylation was monitored by conversion of m^7^G*pppA-RNA→m^7^G*pppAm-RNA. Both methylation assays were performed with 1.5 µM WNV MTase (or 1.5 µM DENV2 MTase or 3 µM DENV3 MTase or 3.2 µM YFV MTase), 80 µM AdoMet, 0.36 µM G*pppA-RNA or m^7^G*pppA-RNA substrate, and various concentrations of each compound. The methylation reactions were digested with nuclease P1 to release cap moieties (m^7^G*pppAm, m^7^G*pppA, and G*pppA). The cap molecules were separated on a thin-layer chromatograph (TLC), and quantified by a PhosphorImager. The percentage of activity was determined after quantification of m^7^G*pppA, m^7^G*pppAm, and G*pppA.

### Biotinylation of MTase

Biotin was conjugated to the WNV and DENV3 MTase using the EZ-Link NHS-biotin Kit (Pierce), according to manufactory protocol. Specifically, the MTases of WNV (30 µM) and DENV3 (65 µM) were dialyzed into phosphate buffered saline (PBS), and mixed with the biotin reagent at a final concentration of 1 mM at 23°C overnight. Unconjugated biotin was removed by FPLC over an HiTrap desalting column (GE HealthCare), and the ratio of conjugated biotin to the DENV3 MTase (13:1) was determined using a Biotin Quantitation kit (Pierce).

### AdoMet binding assay

Biotinylated DENV3 MTase (580 nM) was mixed with the polyvinyltoluene (PVT) scintillation proximity assay (SPA) beads (1.5 mg/ml, PerkinElmer) and the indicated concentrations of AdoMet, AdoHcy, or SIN in the AdoMet Binding Buffer (20 mM Tris pH 7.5, 50 mM NaCl, 10 mM KCl, 2 mM MgCl_2_, 2 mM MnCl_2_, 0.05% CHAPS) in a white 96-well clear-bottom plate. The samples were mixed by gentle rocking for 20 minutes at 23°C, followed by the addition of 1.65 µCi of ^3^H-AdoMet (425 nM) to a final sample volume of 50 µl. After mixing for another 15 minutes at 23°C, samples were then centrifuged for 2 minutes at 500g and analyzed on a Microbeta^2^ 2450 plate counter (PerkinElmer) using the default ^3^H-Scintillation Proximity Assay protocol within the manufactory software. The competitive binding affinities (*k*_*d*_) were determined by fitting of the dose–response curve using the ORIGIN software package (OriginLab Corporation).

### Cytotoxicity assay

Cytotoxicity was measured by a MTT cell proliferation assay using the 3-(4,5-dimethylthiazol-2-yl)-2,5-diphenyl tetrazolium bromide (MTT) method (ATCC). Approximately 2 x 10^4^ human A549 cells (ATCC) in 100 µl of media were seeded into 60 wells of a 96 well plate, the remaining wells held media. Plates were held at room temperature for 1 hour and then incubated for 20-24 hours. The media was removed and 100 µl of media containing decreasing concentrations of antiviral compound in 1% DMSO were added to the wells. All determinations were performed in triplicate. After 42 hours incubation at 37°C, 10 µl of MTT was added to the wells and incubated another 2-4 hours. Detergent (100 µl) was placed in the wells and the plate was incubated for at least 3 hours at room temperature in the dark. A microtiter plate reader (Ely808, BioTek Instruments, Inc.) with a 570 nm filter was used to record absorbance. All determinations were performed in triplicate. After adjusting the absorbance for background and comparing to untreated controls, a dose-response curve was plotted and the cytotoxic concentration CC_50_ (the concentration of inhibitor required to reduce cell viability by 50%) was calculated using nonlinear regression analysis in the ORIGIN software package (OriginLab Corporation).

### Antiviral assay

A viral titer reduction assay was used to determine the compounds effect on WNV. Approximately 2 x 10^5^ human A549 cells in 1.0 ml of media were seeded into each well of a 24 well plate. At 24-30 hours after seeding, dilutions at 2X the desired concentration of the compound were made in 2% DMSO media and 50 µl was added to wells in triplicate. Immediately following, 50 µl of media containing WNV (NY99) or DENV2 (New Guinea C) at a concentration to yield a multiplicity of infection (MOI) 0.1 PFU/cell (PFU, plaque-forming unit), was added to the wells. After one hour incubation at 37°C, 400 µl of media containing the desired concentrations of the compound was added to the each well. After 42 hours incubation at 37°C, culture media was collected, and stored at -80°C for later quantification using a plaque assay. For the plaque assay, Vero cell monolayers in 6-well plates were seeded 3-4 days prior to infection to achieve a confluent monolayer. Dilutions of the viral samples were made and 100µl of each dilution were inoculated into each of 2 wells, rocked gently to distribute virus, and incubated for 1 hour at 37°C. Cells are then overlaid with a nutrient medium containing 0.6% oxoid agar and incubated at37°C. After 2-5 days, depending on the virus a second overlay containing 2% neutral red is added to the cells and then incubated overnight. Plaques are counted daily for 1-2 days until no significant increase is seen.

### Computational Methods

The Molecular Mechanics (MM) program CHARMM [39,40] was used for the explicit solvent molecular dynamics (MD) simulations and their subsequent analysis. The CHARMM22 protein force field [41] with the CMAP correction [42] was used for the protein, the TIP3P model for the water [43], and Beglov and Roux parameters for the potassium and chloride ions [44]. Parameters for AdoHcy were obtained from a previous study [45], and parameters for SIN were generated using CGENFF [46–48] (Table **S2**). These SIN parameters were adjusted to ensure transfer from appropriate chemical contexts. The final parameters used are provided in the supplementary material, where their origin is also annotated.

The WNV MTase structure bound to SIN was used as the starting point for the calculations (PDB ID: 3LKZ, chain A [23]). It was solvated in an 80 Å dimension cubic water box with 38 potassium and 46 chloride ions, representing a 150 mM KCl buffer. The final system consisted of 48487 atoms including 14722 waters. The full system was minimized using 1000 steps of Steepest Descent (SD) and 500 steps of Adopted-Basis Newton Raphson (ABNR) minimization with a convergence cutoff of 0.001 kcal/mol. Long-range electrostatic interactions were treated using the Particle Mesh Ewald (PME) approach [49] with a B-spline order of 4 and a Fast Fourier Transform grid of one point per Å and a real-space Gaussian width kappa of 0.3 Å^−1^. Real space and Lennard-Jones (LJ) interaction cutoffs of 12 Å were used with non-bond interaction lists maintained and heuristically updated out to 16 Å. A constant pressure and temperature (NPT) ensemble [50] was used for the MD simulations.

The main purpose of these simulations was to understand the energetic differences between the binding of the two ligands AdoHcy and SIN to WNV MTase. An ancillary aim was to achieve this analysis through relatively short explicit solvent simulations that could be used as a way to refine binding energy estimations for future inhibitor design. For this purpose, the explicit solvent simulations were limited to a short 100 ps duration, which required 36 CPU hours on a single 2.26 GHz Intel Xeon processor. All protein and ligand non-hydrogen atoms were harmonically restrained with a force constant of 10 kcal/mol/Å^2^ for the first 20 ps increment, 5 kcal/mol/Å^2^ for the second 20 ps increment, 2 kcal/mol/Å^2^ for the third 20 ps increment, 1 kcal/mol/Å^2^ for the fourth 20 ps increment, and finally 0.5 kcal/mol/Å^2^ for the final 20 ps increment. The final weak restraint was kept in place to ensure that the sampling observed was close to the starting crystal structure, but still allow for any necessary relaxation of ligand or protein atoms. One hundred configurations saved every 0.2 ps from these final 20 ps were used for the Molecular Mechanics Poisson-Boltzmann Surface Area (MM-PBSA) analysis [51]. The MM energies were calculated without any cutoff for the non-bonded interactions. The surface area term was calculated using a coefficient of 0.00542 kcal/mol/Å^2^ and a constant B term of 0.92 kcal/mol. For the Poisson-Boltzmann calculations, a grid with spacing of 0.4 Å was overlaid on the solute which extended at least 20 Å from the edges of the solute, and the electrostatic solvation free energy was computed by solving the Poisson-Boltzmann equation using the PBEQ module in CHARMM. A solute dielectric of 4 and a solvent dielectric of 80 was used, and the solute-solvent boundary was estimated using a reentrant surface assuming a water sphere radius of 1.4 Å. The solute entropy was calculated by quasi-harmonic analysis of the 100 snapshots using the vibran module in CHARMM.

## Supporting Information

Figure S1 **NMR parameters for synthesized compounds.**
(DOC)

Table S1 **Atomic contributions for SIN or AdoHcy vacuum interaction with WNV MTase.**
(RTF)

Table S2 **SIN topology and parameters in **CHARMM** format.**
(DOCX)
