# Supplementary material for: S-Adenosyl-Homocysteine Is a Weakly Bound Inhibitor for a Flaviviral Methyltransferase
Source: PLoS One. 2013 Oct 9;8(10):e76900. doi: 10.1371/journal.pone.0076900 (PMC3793912; doi:10.1371/journal.pone.0076900)
Supplement: Figure S1 — NMR parameters for synthesized compounds. (DOC) [file pone.0076900.s001.doc]

**Figure S1, NMR parameters for synthesized compounds**

**GRL-001-12-MT:**

^1^H NMR (400 MHz, D_2_O) δ 8.44 (s, 1H), 8.40 (s, 1H), 5.00 – 4.84 (m, 1H), 4.57 (dd, *J* = 8.7, 6.0 Hz, 1H), 4.08 (dt, *J* = 14.9, 5.6 Hz, 2H), 2.85 (m, 6H), 2.66 – 2.53 (m, 1H), 2.41 – 2.12 (m, 3H), 1.92 (dd, *J* = 22.5, 10.6 Hz, 1H). MS (ESI) m/z 383 (M+H^+^).

**GRL-015-11-MT:**

^1^H NMR (400 MHz, D_2_O) δ 8.45 (s, 1H), 8.40 (s, 1H), 4.53 (dd, *J* = 9.0, 5.8 Hz, 1H), 4.14 – 4.07 (m, 1H), 4.06 – 3.98 (m, 1H), 3.82 – 3.55 (m, 4H), 2.60 – 2.33 (m, 2H), 2.32 – 2.10 (m, 2H), 1.89 (m, 1H). MS (ESI) m/z 367 (M+H^+^).

**GRL-014-11-MT:**

^1^H NMR (400 MHz, D_2_O) δ 8.29 (s, 1H), 8.22 (s, 1H), 4.52 (dd, *J* = 9.3, 5.7 Hz, 1H), 4.26 (dd, *J* = 9.4, 4.3 Hz, 1H), 4.06 (dd, *J* = 5.7, 3.2 Hz, 1H), 3.60 (m, 4H), 2.54 – 2.43 (m, 1H), 2.41 – 2.31 (m, 1H), 2.21 – 2.09 (m, 1H), 1.97 (s, 3H), 1.93 – 1.73 (m, 2H). MS (ESI) m/z 409 (M+H^+^).

**GRL-016-11-MT:**

^1^H NMR (400 MHz, D_2_O) δ 8.23 (s, 1H), 8.20 (s, 1H), 7.47 – 7.28 (m, 5H), 4.56 – 4.46 (m, 1H), 4.11 (m, 1H), 3.83 (dd, *J* = 7.0, 4.4 Hz, 1H), 3.78 – 3.57 (m, 4H), 2.54 – 2.32 (m, 2H), 2.28 – 2.01 (m, 2H), 1.79 (dd, *J* = 21.4, 10.6 Hz, 1H). MS (ESI) m/z 457 (M+H^+^).
