# Supplementary material for: S-Adenosyl-Homocysteine Is a Weakly Bound Inhibitor for a Flaviviral Methyltransferase
Source: PLoS One. 2013 Oct 9;8(10):e76900. doi: 10.1371/journal.pone.0076900 (PMC3793912; doi:10.1371/journal.pone.0076900)
Supplement: Table S1 — Atomic contributions for SIN or AdoHcy vacuum interaction with WNV MTase. (RTF) [file pone.0076900.s002.rtf]

Table S1. Atomic contributions for SIN or AdoHcy vacuum interaction with WNV MTase
Atom Number	Atom name	Average vacuum interaction energy (kcal/mol)	
		AdoHcy	SIN	SIN-AdoHcy	
1	  N	-50.67	-44.91	5.75                                 N	
2	  HT1	44.00	51.55	7.55                                 HT1	
3	  HT2	53.44	49.73	-3.71	
4	  HT3	59.64	44.21	-15.43	
5	  CA	38.67	36.55	-2.12	
6	  HA	17.19	16.70	-0.50	
7	  CB	-36.49	-32.16	4.33	
8	  HB1	18.34	15.98	-2.36	
9	  HB2	18.77	16.36	-2.41	
10	  CG	-25.05	-21.78	3.27	
11	  HG1	14.28	12.61	-1.67	
12	  HG2	15.91	12.26	-3.65	
13	  SD / CD	-16.46	13.91	30.36	
14	  HD	0.00	13.13	13.13	
15	  NE	0.00	-152.39	-152.39	
16	  HE1	0.00	51.56	51.56	
17	  HE2	0.00	57.54	57.54	
18	  C5	-19.69	-18.16	1.53	
19	  H5	13.50	11.30	-2.20	
20	  H5	12.61	10.80	-1.81	
21	  C	75.91	70.99	-4.93	
22	  OT1	-170.12	-156.11	14.01	
23	  OT2	-166.86	-154.40	12.47	
24	  C4	21.47	19.45	-2.02	
25	  H4	12.36	11.40	-0.96	
26	  O4	-70.18	-62.07	8.11	
27	  C1	20.17	17.63	-2.54	
28	  H1	11.48	10.90	-0.58	
29	  N9	-7.05	-6.75	0.30	
30	  C5	31.98	21.66	-10.32	
31	  N7	-87.95	-57.93	30.02	
32	  C8	43.08	28.77	-14.31	
33	  H8	15.90	10.11	-5.79	
34	  N1	-75.19	-67.69	7.50	
35	  C2	51.69	45.26	-6.42	
36	  H2	12.41	11.78	-0.63	
37	  N3	-88.25	-76.98	11.27	
38	  C4	50.67	38.47	-12.20	
39	  C6	48.03	35.55	-12.49	
40	  N6	-78.91	-59.75	19.16	
41	  H61	34.85	29.33	-5.52	
42	  H62	40.25	28.53	-11.72	
43	  C2	16.91	15.85	-1.06	
44	  H2	11.52	9.55	-1.97	
45	  O2	-74.75	-86.27	-11.52	
46	  H2	47.73	49.91	2.18	
47	  C3	17.29	17.26	-0.03	
48	  H3	11.38	11.00	-0.38	
49	  O3	-80.45	-101.11	-20.67	
50	  H3T	42.15	64.02	21.87	
					
Total		-124.50	-136.88	-12.38	
