# Supplementary material for: S-Adenosyl-Homocysteine Is a Weakly Bound Inhibitor for a Flaviviral Methyltransferase
Source: PLoS One. 2013 Oct 9;8(10):e76900. doi: 10.1371/journal.pone.0076900 (PMC3793912; doi:10.1371/journal.pone.0076900)
Supplement: Table S2 — SIN topology and parameters in CHARMM format. (DOCX) [file pone.0076900.s003.docx]

**Table S2, SIN topology and parameters in CHARMM format**

**Topology**

Patch to be applied to residue ADE to generate SIN (to be applied only after applying the 3TER patch):

PRES SIN 0.00

DELETE ATOM P

DELETE ATOM O1P

DELETE ATOM O2P

DELETE ATOM O5’

GROUP

ATOM N NH3 -0.30

ATOM HT1 HC 0.33

ATOM HT2 HC 0.33

ATOM HT3 HC 0.33

ATOM CA CT1 0.21

ATOM HA HB 0.10

GROUP

ATOM CB CT2 -0.18

ATOM HB1 HA 0.09

ATOM HB2 HA 0.09

GROUP

ATOM CG CT2 -0.14

ATOM HG1 HA 0.09

ATOM HG2 HA 0.09

ATOM CD CT1 0.10

ATOM HD HB 0.09

ATOM NE NH2 -0.96

ATOM HE1 H 0.34

ATOM HE2 H 0.34

ATOM C5’ CN8B -0.13

ATOM H5’ HN8 0.09

ATOM H5” HN8 0.09

GROUP

ATOM C CC 0.34

ATOM OT1 OC -0.67

ATOM OT2 OC -0.67

GROUP

ATOM C3’ CN7 0.14

ATOM H3’ HN7 0.09

ATOM O3’ ON5 -0.66

ATOM H3T HN5 0.43

BOND C OT1

BOND N CA

BOND CB CA CG CB CD CG C5’ CD C CA

BOND CA HA CB HB1 CB HB2 CG HG1 CG HG2

BOND CD HD CD NE NE HE1 NE HE2

BOND HT1 N HT2 N HT3 N OT2 C

IMPR OT1 CA OT2 C

**Parameters**

Bond, angle, and dihedral parameters in CHARMM format required for SIN:

**BONDS**

CN8B CT1 222.5 1.512 ! from CN7 CN8B bond parameter

**ANGLES**

HB CT1 CN8B 34.5 110.1 22.53 2.179 ! from HN7 CN7 CN8 angle parameter

CT2 CT1 CN8B 45.0 110.0 ! from CN7 CN7 CN8B angle parameter

NH2 CT1 CN8B 50.0 107.0 ! from NH2 CT1 C angle parameter

CT1 CN8B HN8 33.43 110.1 22.53 2.179 ! from HA CT2 CT1 angle parameter

CT1 CN8B CN7 58.35 113.50 11.16 2.561 ! from CT2 CT2 CT1 angle parameter

**DIHEDRALS**

CT2 CT1 CN8B HN8 0.195 3 0.0 ! from HN8 CN8B CN8 CN7 dihedral parameter

CT2 CT1 CN8B CN7 0.5 4 0.0 ! from CN7 CN7 CN8 CN8B dihedral parameter

CT2 CT1 CN8B CN7 0.1 3 0.0 ! dihedral parameter

CT1 CN8B CN7 CN7 0.5 4 0.0 ! from CN7 CN7 CN8 CN8B dihedral parameter

CT1 CN8B CN7 CN7 0.1 3 0.0 ! dihedral parameter

CT1 CN8B CN7 HN7 0.195 3 0.0 ! from CN8B CN8 CN7 HN7 dihedral parameter

CT1 CN8B CN7 ON6B 3.4 1 180.0 ! from ON6B CN7 CN8B ON5 dihedral parameter

HB CT1 CN8B CN7 0.195 3 0.0 ! from HN8 CN8B CN7 CN7 dihedral parameter

HB CT1 CN8B HN8 0.195 3 0.0 ! from HN7 CN7 CN8B HN8 dihedral parameter

NH2 CT1 CN8B CN7 0.000 1 0.0 ! from NH1 C CT1 CT2 dihedral parameter

NH2 CT1 CN8B HN8 0.000 3 0.0 ! from NH1 C CT2 HA dihedral parameter

H NH2 CT1 CN8B 0.110 3 0.0 ! from H NH2 CT1 CT2 dihedral parameter
